# High-throughput sequencing and analysis of microbial communities in the mangrove swamps along the coast of Beibu Gulf in Guangxi, China

**DOI:** 10.1038/s41598-019-45804-w

**Published:** 2019-06-28

**Authors:** Bin Gong, Hongming Cao, Chunyan Peng, Vanja Perčulija, Guixiang Tong, Huaiyi Fang, Xinxian Wei, Songying Ouyang

**Affiliations:** 1 Guangxi Key Laboratory of Marine Disaster in the Beibu Gulf, Beibu Gulf University, Qinzhou, 535000 China; 2 The Key Laboratory of Innate Immune Biology of Fujian Province, Provincial University Key Laboratory of Cellular Stress Response and Metabolic Regulation, Biomedical Research Center of South China, Key Laboratory of OptoElectronic Science and Technology for Medicine of Ministry of Education, College of Life Sciences, Fujian Normal University, Fuzhou, 350117 China; 3 Guangxi Key Laboratory of Aquatic Genetic Breeding and Healthy Aquaculture, Academy of Fishery Sciences, Nanning, Guangxi 530021 China

**Keywords:** Microbiome, Microbial ecology

## Abstract

Mangrove swamp is one of the world’s richest and most productive marine ecosystems. This ecosystem also has a great ecological importance, but is highly susceptible to anthropogenic disturbances. The balance of mangrove ecosystem depends largely on the microbial communities in mangrove sediments. Thus, understanding how the mangrove microbial communities respond to spatial differences is essential for more accurate assessment of mangrove ecosystem health. To this end, we performed the first medium-distance (150 km) research on the biogeographic distribution of mangrove microbial communities. The hypervariable regions of 16S rRNA gene was sequenced by Illumina to compare the microbial communities in mangrove sediments collected from six locations (i.e. Zhenzhu harbor, Yuzhouping, Maowei Sea, Qinzhou harbor, Beihai city and Shankou) along the coastline of Beibu Gulf in Guangxi province, China. Collectively, *Proteobacteria*, *Bacteroidetes*, *Chloroflexi*, *Actinobacteria*, *Parvarchaeota*, *Acidobacteria* and *Cyanobacteria* were the predominant phyla in the mangrove sediments of this area. At genus level, the heat map of microbial communities reflected similarities between study sites and was in agreement with their biogeographic characteristics. Interestingly, the genera *Desulfococcus*, *Arcobacter*, *Nitrosopumilus* and *Sulfurimonas* showed differences in abundance between study sites. Furthermore, the principal component analysis (PCA) and unweighted UniFrac cluster tree of beta diversity were used to study the biogeographic diversity of the microbial communities. Relatively broader variation of microbial communities was found in Beihai city and Qinzhou harbour, suggesting that environmental condition and historical events may play an important role in shaping the bacterial communities as well. This is the first report on medium-distance range distribution of bacteria in the mangrove swamp ecosystem. Our data is valuable for monitoring and evaluation of the impact of human activity on mangrove habitats from the perspective of microbiome.

## Introduction

Mangrove swamps are located along the coastlines of tropical and subtropical seas around the world. As one of the world’s four most productive marine ecosystems, mangrove swamps host a large variety of species^1^. In China, mangrove swamps are mainly found in the southeastern provinces, including Hainan, Guangxi, Guangdong, Fujian and Taiwan. The largest mangrove communities are in Guangxi, where 15 mangrove species have been documented in mangrove swamps covering a total area of about 8374.9 km^2 ^^2,3^. For this reason, two National Mangrove Reserves have already been established in Guangxi province: Shankou Mangrove Ecological National Nature Reserve and Beilun Estuary Nature Reserve.

Mangrove ecosystems are economically important, providing highly valuable commercial products as well as fishery and aquaculture resources^4^. Mangroves also have enormous ecological value, participating in protection and stabilization of coastlines, purification of marine environment^5^ and fixation of organic carbon^6,7^. However, numerous studies indicate that mangrove ecosystems have suffered on a global scale from human activities such as deforestation^8^, sewage disposal^9^, oil spills and petroleum production^10^. Anthropogenic disturbances have resulted in a significant loss of world’s mangrove swamps^11–13^. Therefore, it is of great importance to systematically investigate and evaluate the health status of mangrove ecosystems.

In recent years, researchers have been paying more and more attention to the ecosystem health of mangrove habitats in Beibu Gulf of Guangxi, China. To assess the health of the mangrove ecosystems in this area, many environmental factors and sentinel organisms have been used as indicators, such as fish diversity^14^, heavy metal pollution^15–17^, organic contamination^18^, benthic macroinvertebrates^19^ and change of mangrove landscape pattern^20^. Nevertheless, more information is needed for a more profound, systematic and comprehensive estimation of mangrove ecosystem health.

An important contribution to the understanding of microbial composition and diversity in mangrove swamps was made by Dos Santos *et al*. and Wu *et al*. who used next-generation sequencing to analyze the microbial composition and function in mangrove ecosystems^21,22^. Microbial communities in mangrove sediments play an essential role in mangrove ecosystems. These microbes are particularly important in global biogeochemical recycling of carbon, nitrogen, phosphorus and sulfur in mangrove swamps^23^. Some microbial species inhabiting mangrove root zones promote mangrove growth by participating in nitrogen fixation and solubilizing of phosphorus^24,25^. Microbes are also responsible for degradation of pollutants in mangrove ecosystems^26^. In addition, the microbes present in mangrove sediments are sensitive indicators of environmental change. The structure of the mangrove microbial community changes in response to the variations in forest type^27^, water salinity^28^, flooding^29^, pollution^30^, nutrient condition^31,32^ etc.

Here, our aim was to understand how the mangrove microbial communities respond to spatial differences on a medium-distance scale (distance of up to 150 km). By using an Illumina sequencing platform, we sought to analyze and compare the mangrove microbial communities at six different mangrove habitats located along the coastline of Beibu Gulf in Guangxi, China. The study aimed to answer the following two questions: (1) what are the differences between microbial communities from the six different study sites with regard to their structure, abundance and diversity of microbes? and (2) is there any relationship between the structure of microbial communities and geographical position of mangrove swamps?

## Materials and Methods

### Study sites and sampling

**Figure 1:**
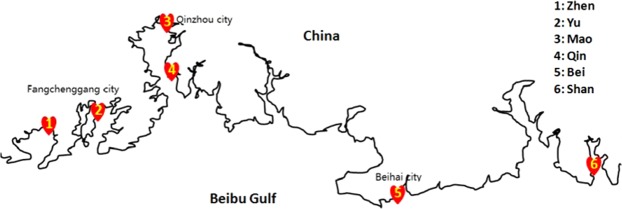
Map of survey area in the northern Beibu Gulf, Guangxi province, China. The enumerated red symbols indicate the location of the six sampling stations.

Nineteen sediment samples were collected at six different mangrove habitats (study sites) in the northern Beibu Gulf of Guangxi province in October 2016. The sediment sample (the top 10–20 cm of sediment) was collected with aseptic plastic bags at each sampling point (defined as a location where a sample was obtained within a study site). The sampling points were limited to the high tide and boundaries of mangrove swamps. Three to four samples were collected at each study site, with distance of at least 300 meters between the sampling points. All the samples were homogenized, pooled and immediately stored at −20 °C. From west to east, the six study sites (with the collected samples indicated in brackets) were Zhenzhu harbor (Zhen 1–3), Yuzhouping (Yu 1–3), Maowei Sea (Mao 1–3), Qinzhou harbor (Qin 1–3), Beihai city (Bei 1–3) and Shankou (Shan 1–4) (Fig. 1). Site Zhen is located in the Beilun Estuary Nature Reserve, a well-protected area containing 15 species of mangrove trees belonging to 11 families^33^. Site Yu (sandy soil) is located in Fangchenggang, a coastal city and a major port of Guangxi province. Site Mao is located in the estuary of Qinjiang River, the main river flowing through the city of Qinzhou. Site Qin is located in Qinzhougang, a big and fast-developing port specialized in heavy industry. Site Bei is located in a mangrove-growing zone of Beihai city. The area is characterized by sandy soil and a nearby sea duck farm and a shrimp pond. Site Shan is located at Shankou Mangrove Ecological National Nature Reserve, another well-protected reserve with diverse and abundant primordial mangrove forests. The character of sampling sites of mangrove habitats in our present study were indicated below (Table 1).

**Table 1:** Characteristics of mangrove habitats where sampling was conducted in the present work.

Code	Geographical location name	Longitude and latitude	Conservation status	Natural condition	Soil condition
Zhen	Zhenzhu harbor, Beilun Estuary Nature Reserve	N 21°36′ E 108°14′	Well protected	Far from city and agricultural activities	muddy soil
Yu	Yuzhouping	N 21°38′ E 108°22′	Not	Near city and big port	Sandy soil
Mao	At the estuary of Qinjiang River into Maowei Sea	N 21°52′ E 108°34′	Not	Far from city	muddy soil
Qin	Qinzhou harbor	N 21°44′ E 108°35′	Not	Near petrochemical plant and human residential area	muddy soil
Bei	Beihai city	N 21°25′ E 109°11′	Not	Near city, an aquatic farm and sea duck farming	Sandy soil
Shan	Shankou Mangrove Ecological National Nature Reserve	N 21°29′ E 109°45′	Well protected	Far from city and agricultural activities	muddy soil

### DNA extraction, PCR amplification, library preparation and sequencing

The DNA was extracted using an GenElute™ Soil DNA Isolation Kit (DNB100, Sigma-Aldrich, China). Afterwards, total DNA was purified and concentrated. The DNA samples were further diluted to 1 ng/μL with sterile water, and were then analyzed by agarose gel electrophoresis and quantified by NanoDrop™ 2000 spectrophotometer. The universal primers 341F (5′-CCTACGGGNGGCWGCAG-3′) and 909R (5′-GACTACHVGGGTATCTAATCC-3′) were used to target the V3-V4 hypervariable regions of bacterial 16S rRNA gene^22,34^. The PCR amplification was carried out with 2× Taq PCR Master Mix (Solarbio^®^ LIFE SCIENCES, China). The PCR products were analyzed by electrophoresis using 2% agarose gel. Samples with bright main strip between 400–450 bp were chosen for further experiments. PCR products were purified with Qiagen Gel Extraction Kit (Qiagen, Germany). Sequencing libraries were generated using TruSeq® DNA PCR-Free Sample Preparation Kit (Illumina, USA). The library quality was assessed on the Qubit@ 2.0 Fluorometer (Thermo Scientific) and Agilent Bioanalyzer 2100 system. Lastly, the libraries were sequenced by Illumina HiSeq 2500 System.

### Bioinformatics and data analysis

The raw data sequences were assigned to samples according to their unique indices. The 16S rDNA primers and indices were then cleaved to generate paired-end reads. The paired-end reads were merged using FLASH (V1.2.7)^35^; the raw tags were filtered to obtain the high-quality clean tags using QIIME software package (V1.7.0)^36^. Sequence analysis was performed in UPARSE (V7.0.1001)^37^. Sequences with ≥97% similarity were assigned to same operational taxonomic units (OTUs). For each representative sequence, the Greengenes Database was used to annotate taxonomic information^38^. Sequence data have been deposited in the National Center for Biotechnology Information (NCBI) Sequence Read Archive (SRA) under the accession numbers SRR8696312, SRR8696313, SRR8696314, SRR8696315, SRR8696316, SRR8696317, SRR8696318, SRR8696319, SRR8696320, SRR8696321, SRR8696322, SRR8696323, SRR8696324, SRR8696325, SRR8696326, SRR8696327, SRR8696328, SRR8696329 and SRR8696330.

In order to determine alpha diversity, four metrics were calculated: Chao1 metric (estimating the species richness), abundance-based coverage estimator (ACE, similar to Chao1), simpson and Shannon diversity index. Beta diversity was generated using the OTUs table (including all the taxonomic units) to evaluate differences in complexity of species between samples. Cluster analysis was preceded by principal component analysis (PCA) to compare the structure and composition of the whole bacterial and archaea communities in nineteen sediment samples using the FactoMineR package and ggplot2 package in R software (Version 2.15.3)^39,40^. Unweighted Pair-group Method with Arithmetic Means (UPGMA) clustering was conducted using the QIIME software (Version 1.7.0), and a hierarchical clustering was constructed to interpret the distance matrix based on average linkage^41^.

### Statistical analysis

Data were compiled and transformed in Microsoft Excel. The analysis of similarities (ANOSIM) for the unweighted UniFrac distances and the Multi Response Permutation Procedure (MRPP) for the PCA analysis between factors was conducted in QIIME using the Vegan package. Other statistical tests were performed using the software SigmaStat, version 2.01 (Jandel Scientific, San Rafael, CA, USA). Values of p < 0.05 were considered statistically significant.

## Results

### Amplicon analysis by illumina sequencing

A total of 1,843,133 raw 16S rRNA V3-V4 sequences were obtained from 19 sediment samples at six study sites along the coastline of Beibu Gulf in Guangxi, China (Fig. 1). The numbers of taxon tags ranged from 21,244 to 66, 204 among the samples, with Qin2 having the largest number of OTUs (6,202) and Mao1 having the smallest number of OTUs (3,330) (Table 2). Rarefaction curves, Chao1 richness, ACE (Abundance-based Coverage Estimator) metric, Simpson’s and Shannon’s diversity indices were calculated for analysis of alpha diversity (Table 2). Shannon’s diversity index indicated that the sequencing depth was sufficient to capture the microbial diversities in all samples. The values of Shannon’s diversity index and ACE metric both indicated that Zhen and Qin possessed greater species richness than Shan (one-way ANOVA, P < 0.05), where the values of Chao1 demonstrated that Qin possessed greater species richness than Shan (one-way ANOVA, P < 0.05). According to Shannon’s diversity index, the species richness of Bei (SD = 0.42) and Yu (SD = 0.29) displayed the biggest standard deviation when compared to Zhen (SD = 0.18), Shan (SD = 0.23), Qin (SD = 0.13) and Mao (SD = 0.12). Moreover, the standard deviation of Chao1 and ACE metric in Mao (SD = 110.7 and SD = 74.5, respectively) was smaller than that of other groups (SD values ranging from 441.8 to 904.0 for Chao1 and 451.5 to 1040.1 for ACE).

**Table 2:** Alpha diversity indicators of the microbes from sediment samples collected at six mangrove swamp study sites along the coastline of Beibu Gulf in Guangxi, China.

Sample name	OTU number	Shannon	Simpson	Chao1	ACE
Qin1	4245	10.21	0.997	4783.79	4831.50
Qin2	6202	10.14	0.996	5804.84	6168.07
Qin3	5248	10.45	0.997	6037.55	6378.51
Bei1	3591	9.44	0.993	4044.15	4063.70
Bei2	5735	10.46	0.998	5679.11	5885.49
Bei3	4805	9.89	0.996	4491.82	4857.99
Mao1	3330	9.91	0.997	4774.09	5111.95
Mao2	5132	10.18	0.997	5008.09	5236.32
Mao3	5573	9.94	0.995	5009.95	5289.81
Shan4	4008	9.59	0.995	3961.10	4035.52
Shan5	3615	9.44	0.995	3595.18	3790.32
Shan6	5432	9.99	0.995	4808.27	4997.26
Shan7	3832	9.95	0.997	4214.14	4231.59
Yu1	4259	9.64	0.994	3978.91	4184.62
Yu2	5408	10.22	0.997	5057.84	5360.45
Yu3	5519	10.30	0.998	5541.97	5807.72
Zhen1	4327	10.03	0.997	4084.09	4245.38
Zhen2	6075	10.46	0.998	6241.61	6714.97
Zhen3	5632	10.37	0.998	5594.54	6022.74

### Taxonomic assignment of the bacterial and archaea communities in mangrove swamps of the northern Beibu Gulf in Guangxi

**Figure 2:**
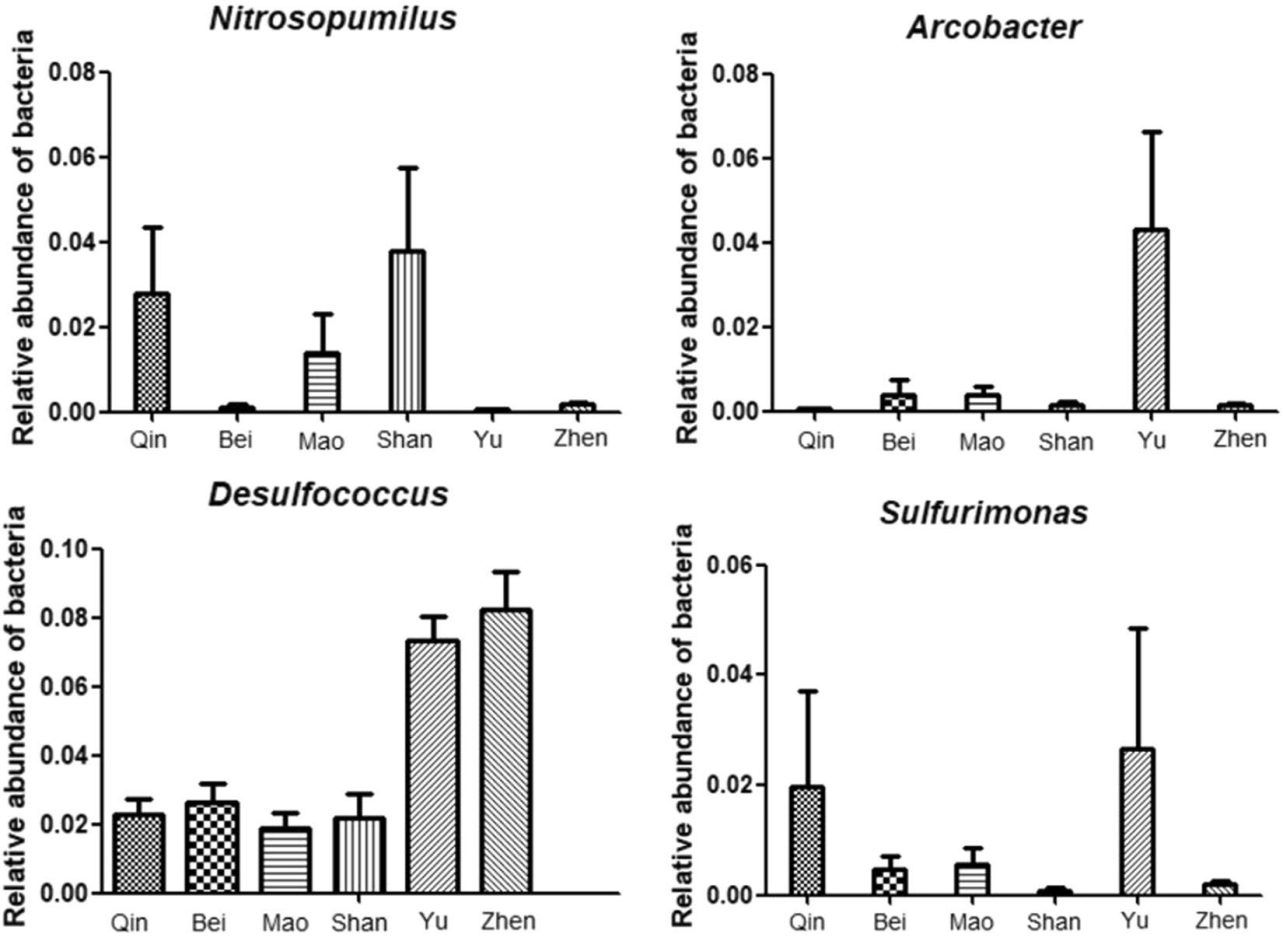
Relative abundance of *Nitrosopumilus*, *Sulfurimonas*, *Desulfococcus* and *Arcobacter* at six study sites.

According to the sequencing results, less than 3.5% OTUs could not be assigned into any known phyla. The other OTUs could be classified into sixty-nine phyla. Of them, the phyla *Proteobacteria*, *Parvarchaeota*, *Chloroflexi*, *Bacteroidetes*, *Cyanobacteria*, *Actinobacteria*, *Acidobacteria*, *Crenarchaeota*, *Firmicutes*, *Euryarchaeota*, *Gemmatimonadetes*, *Nitrospirae*, *Verrucomicrobia*, *Caldithrix*, *Fusobacteria*, *Chlorobi*, *Spirochaetes* were present in all samples. Based on average abundance analysis, *Proteobacteria* was the most predominant phylum (52.3%), followed by *Bacteroidetes* (7.73%), *Chloroflexi* (6.09%), *Actinobacteria* (5.02%), *Parvarchaeota* (4.10%), *Acidobacteria* (3.98%) and *Cyanobacteria* (2.38%). At class level, *Deltaproteobacteria* (19.29%), *Gammaproteobacteria* (18.15%), *Alphaproteobacteria* (10.18%) and *Parvarchaea* (4.08%) were the four largest classes, accounting for 41.62% of the taxon tags. Other abundant classes included *Acidimicrobiia* (3.92%), *Bacteroidia* (3.13%), *Epsilonproteobacteria* (2.80%), *Dehalococcoidetes* (2.74%), *Anaerolineae* (2.50%) and *Chloroplast* (1.84%). At family level, the ten most dominant families were *Desulfobacteraceae* (5.75%), *Desulfobulbaceae* (3.41%), *Rhodobacteraceae* (2.45%), *Helicobacteraceae* (1.65%), *Desulfarculaceae* (1.72%), *Cenarchaeaceae* (1.38%), *Vibrionaceae* (1.01%), *Oceanospirillaceae* (0.96%), *Campylobacteraceae* (0.89%) and *Alteromonadaceae* (0.79%). At genus level, the abundance of *Desulfococcus* at Yu (7.35%) and Zhen (8.24%) was notably higher than the other sites (0.31–3.56%) (P < 0.01), and *Arcobacter* was more represented at Yu (4.28%) in comparison to the other sites (0.009–0.6%) (P < 0.05). Moreover, *Nitrosopumilus* was significantly more abundant in Shan (2.99%) than Bei, Yu and Zhen, where it amounted for 0.009–0.2% of the total microbial population) (P < 0.05). *Sulfurimonas* was litter more abundant at Yu (2.65%) and Qin (1.96%) than the other sites (0.07–0.55%) (Fig. 2).

### Structural comparison of bacterial and archaea communities from different mangrove swamps

**Figure 3:**
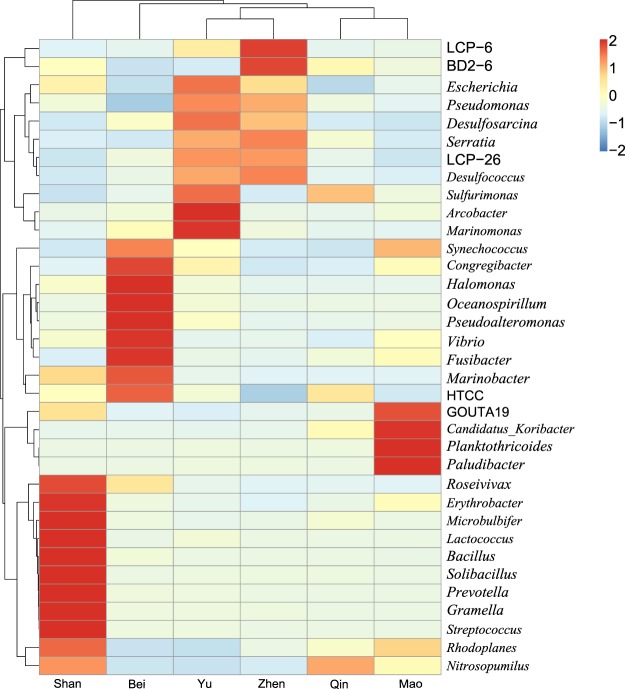
The heat map plot depicts abundance of bacterial community at the genus level among six different mangrove swamps. The relative values are depicted by color intensity with the legend indicated in the upper right corner.

In total, 392 genera of bacteria and archaea were identified in this study. The thirty-five most abundant genera were used to draw a heat map diagram (Fig. 3). As shown in the heat map, eleven genera were present at higher proportion at study site Shan: *Roseivivax*, *Erythrobacter*, *Microbulbifer*, *Lactococcus*, *Bacillus*, *Solibacillus*, *Prevotella*, *Gramella*, *Streptococcus*, *Rhodoplanes* and *Nitrosopumilus* (P < 0.05). Eight known genera (*Synechococcus*, *Congregibacter*, *Halomonas*, *Oceanospirillum*, *Vibrio*, *Pseudoalteromonas*, *Fusibacter* and *Marinobacter*) and an unclassified genus HTCC were comparatively more abundant at site Bei (P < 0.05). Both site Yu and site Zhen were characterized by higher concentration of six genera, namely *Escherichia*, *Serratia*, *Pseudomonas*, *Desulfosporosinus*, LCP-26 and *Desulfococcus* (P < 0.05). In addition, the abundance of the genera *Sulfurimonas*, *Arcobacter* and *Marinomonas* was notable at site Yu, whereas the genera LCP-6 and BD2-6 were highly represented at site Zhen (P < 0.05). The genera *Candidatus*, *Koribacter*, *Planktothricoides*, *Paludibacter* and GOUTA19 were more abundant at site Mao when compared to the other sites (P < 0.05). The structures of microbial communities shown in the heat map indicated higher degree of similarity between sites Qin and Mao, and sites Zhen and Yu. Altogether, cluster analysis showed that similarities between the microbial communities from the six study sites were in good agreement with their biogeographic characteristics.

**Figure 4:**
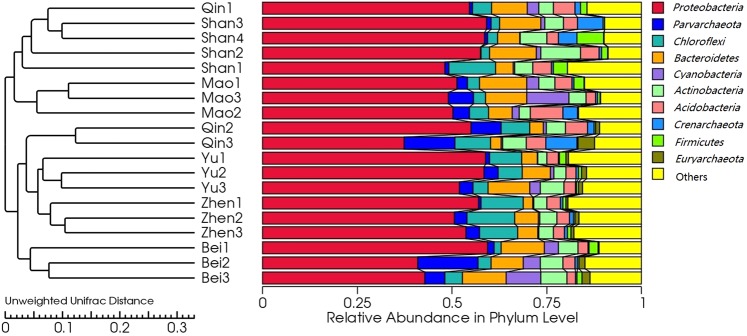
Unweighted uniFrac cluster tree (on the left side) of bacterial and archaea communities was generated using the OTUs table from six different mangrove habitats in Guangxi province, China. A higher degree of similarity was observed between samples collected at the same study site, but different sampling points (ANOSIM R = 0.249, P < 0.05).The histogram of relative abundance on bacterial and archaea communities based on phylum-level is on the right, whicn was used to show their relationship better.

UniFrac was used to determine the structural differences of microbial communities originating from 19 different mangrove sediment samples. Jackknifed UPGMA (Unweighted Pair Group Method with Arithmetic Mean) clustering was performed to cluster the samples based on distance matrix. Strikingly, the unweighted UniFrac cluster tree demonstrated that nineteen microbial communities from mangrove sediments clustered according to their geographic features. We can thus conclude that geography plays a significant role in structuring microbial communities, which is why there is also higher degree of similarity between samples collected at the same study site, but different sampling points (ANOSIM R = 0.249, P = 0.012). Unweighted UniFrac cluster tree showed that microbial community samples from Bei (Bei 1, 2 and 3) sediments cluster in the same branch. Similarly, microbes retrieved from Zhen, Yu, Mao and Shan sediments together form a distinct group as determined by UPGMA. Only microbial communities from site Qin form a discrete cluster (Fig. 4).

**Figure 5:**
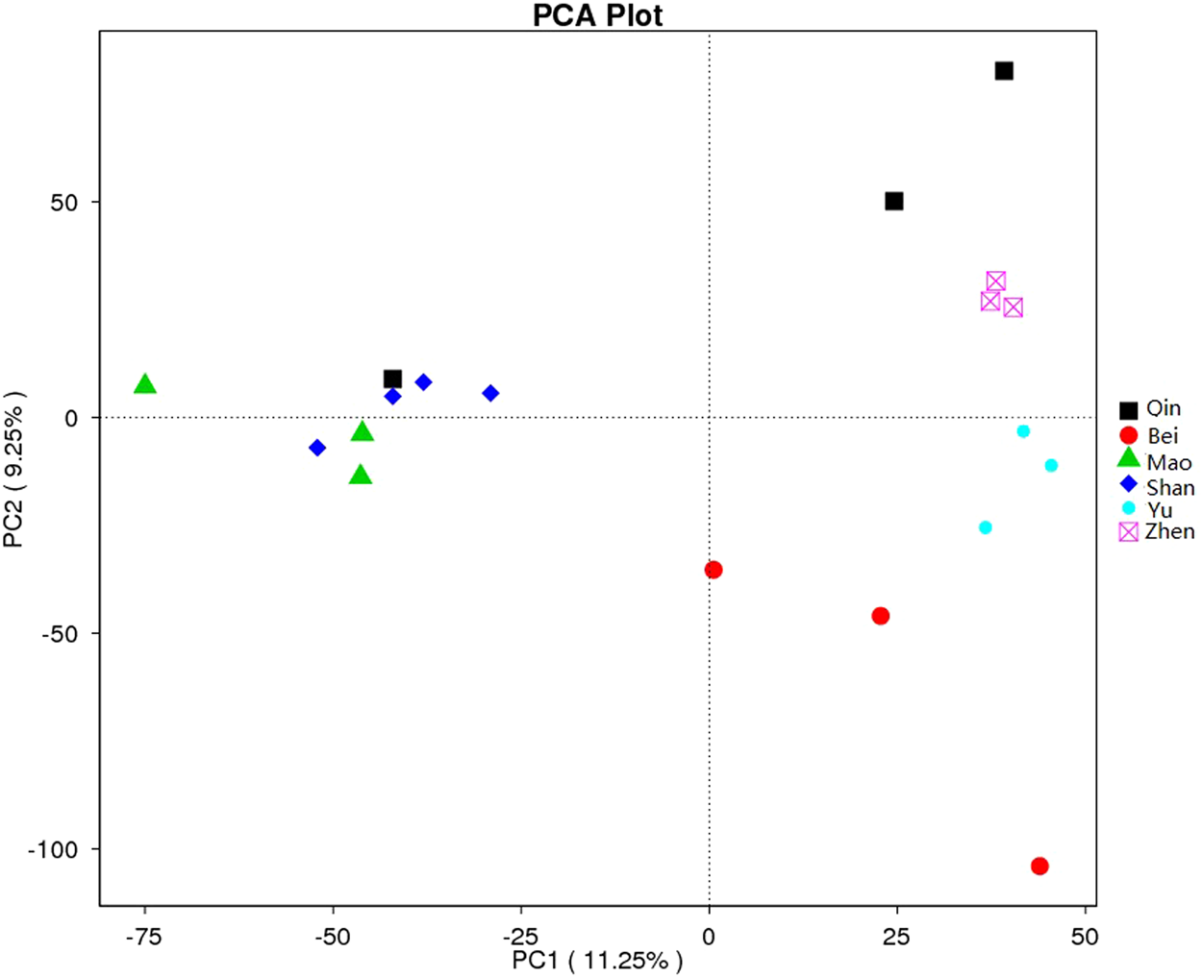
Unweighted UniFrac-based Principal Component Analysis (PCA) of bacterial and archaea communities profiled using 16S rRNA gene sequencing. Each point corresponds to a sample colored according to the corresponding mangrove study site. The legend indicated to the right of PCA plot shows the study sites and their respective colors within the plot. The samples from the same study sites can be seen closer to each other (MRPP, A = 0.1497, Observed delta = 0.1505, Expected delta = 0.177, P < 0.01).

Principal Component Analysis (PCA) plots based on unweighted UniFrac distance metrics were drawn to compare microbial communities from the six mangrove study sites. PC1 and PC2 accounted for 11.25% and 9.25% of the total variation, respectively. PCA plot exhibited an obvious pattern of distribution, with microbial communities from seven mangrove sediments sampled at Shan and Mao, and six from Zhen and Yu displaying closer relative distances. Conversely, the microbial communities from sites Qin and Bei demonstrated higher divergence. In line with the UniFrac cluster tree, PCA analysis separated the samples from six mangrove locations into different groups, with samples from the same study sites being closer to each other (MRPP, A = 0.1497, Observed delta = 0.1505, Expected delta = 0.177, P = 0.009). More specifically, PCA plots revealed relatively tight clustering of samples from locations Zhen, Yu, Mao and Shan, and a relatively broader variation in the samples from Bei and Qin (Fig. 5). This suggests that geographical location is the primary factor shaping the microbial composition, and environmental condition is a secondary factor important for the stability of microbial communities.

## Discussion

Mangrove swamp is a complex ecosystem characterized by soft deposited silt and dense forest, making sampling within the swamp difficult. The sampling was limited to high tide and boundaries of the six mangrove habitats involved in this study, thus lacking a more extensive and comprehensive investigation of microbial distribution patterns in the mangrove swamps along the Beibu Gulf in Guangxi, China. Another limitation of the present work is the sample size is small. However, because the samples were randomly collected from sampling sites, this issue would not affect our conclusion on the two questions that the study aimed to answer.

In the present work, *Proteobacteria*, *Bacteroidetes*, *Chloroflexi*, *Actinobacteria*, *Parvarchaeota*, *Acidobacteria* and *Cyanobacteria* were found to be the most predominant phyla in the mangrove sediments at all six study sites. Such results are in accordance with another study^34^. Similarly, *Proteobacteria* was the most abundant group in mangrove sediments in a number of related researches^34,42–44^. Higher relative abundance of *Acidobacteria* and *Actinobacteria* were associated with the protected mangrove regions, whereas *Chloroflexi* and *Bacteroidetes* were found more abundant at the unprotected sites^42^. Moreover, it was observed that higher distribution of *Actinobacteria* and *Acidobacteria* inclined towards the nutrient-rich inner mangrove sediments, while *Proteobacteria* was more abundant in the outer mangrove sediments^34^. The higher abundance of *Proteobacteria*, especially the classes *Gammaproteobacteria* and *Deltaproteobacteria*, was beneficial for detoxification of pollutants in mangrove soils^45^. *Cyanobacteria* were oxygenic photosynthetic bacteria that are widespread in mangrove swamps, where they were mostly involved in nitrogen fixation and carbon cycle^46,47^. *Parvarchaeota*, which were usually found in extreme environments such as hot springs, likely take part in carbon and nitrogen cycling by degrading multiple saccharides and proteins, and produce ATP via aerobic respiration and fermentation^48^. Many *Desulfococcus* strains were anaerobic sulfate-reducing bacteria (SRB), and some participate in anaerobic degradation of organic compounds^49,50^. *Arcobacter* species were pathogenic bacteria that can infect humans and animals^51^. *Nitrosopumilus*, a genus of ammonia-oxidizing archaea (AOA), were ubiquitous in marine environments and play an important role in the biogeochemical cycles of carbon and nitrogen^52,53^. *Sulfurimonas*, a genus of sulfur-oxidizing *Epsilonproteobacterium*, participated in chemolithotrophic denitrification by oxidizing sulfide to sulfate using nitrate as electron acceptor^54,55^.

Our study found a relatively high abundance of the microbial genera *Sulfurimonas*, *Arcobacter* and *Desulfococcus* at study sites Yu and Zhen. With that in mind, we speculate that site Yu, where a large port has been active for several decades (Table 1), may have accumulated comparatively large concentrations of sulfur and hydrocarbons originating from petroleum contamination. This pollution may have spread all the way to the mangrove nature reserve at site Zhen, which would explain the presence of *Desulfococcus* at this site. The study site Yu was also notable for large presence of the *Arcobacter* genus (family *Campylobacteraceae*). In addition, a high proportion of *Epsilonproteobacteria*, particularly *Helicobacteriaceae* and *Campylobacteraceae*, were detected in our work. All of these taxa are known to harbour famous human or animal pathogens and some were identified as etiologic agents in outbreaks and sporadic cases of gastroenteritis^56^. There was a strong link between *Helicobacter* species and human gastric carcinogenesis, extragastric disease and other disease^57^. In natural environment, *Helicobacter* occurred more frequently in water than in soil^58,59^. *Helicobacteriaceae* has been detected as the most abundant family of sulfuroxidizing bacteria (SOB) in wastewater treatment plants^60^. Many species of the genus *Arcobacter* were found in both marine animal and environmental sources. Pathogenic *Arcobacter* has been isolated from copepods^61^, zooplankton^62^, shellfish^63^, mussels^64^, sea snail *Haliotis gigantea*^65^, seawater^66^ and estuarine sediment^67^, i.e. One reason for their presence in marine environment may be correlated with high levels of fecal pollution^68^. This was in accord with fact that the side Yu is in vicinity of a city (Table 1), and domestic sewage outlets may be located nearby. Moreover, the highly anaerobic character and abundance of sulfur in mangrove sediments are suitable for the proliferation of these kind of bacteria^69^, which is why they are the most predominant taxa in some sampling sites. The presence of potentially pathogenic taxa in mangrove sediment may have ecological and epidemiological implications^62^, and indicates a potential ecological disaster in the near past. We speculated that several death events of marine species occurred in this area may have some correlation with the presence of potentially pathogenic taxa^70^.

The global distribution of microbes can be well described by the Baas Becking hypothesis: “Everything is everywhere, but the environment selects”. In other words, environment is the key factor that determines the structure of microbial community in certain habitat, whereas geological barriers are irrelevant^71^. With regard to marine ecosystems, factors such as temperature^72,73^, water salinity^74^ and pH value^75^ have been reported as main determinants governing the geographical distribution of microorganisms in marine ecosystems. However, little information is known about the biogeographic patterns of oceanic microbial communities. In our study, the heat map of microbial genera demonstrated that structural similarities among the microbial communities from the six mangrove study sites accorded well with their biogeographic characteristics. Moreover, the unweighted UniFrac cluster tree also indicated that geography plays a significant role in structuring microbial communities since the highest degree of similarity exists between the communities from the same study sites. In line with the above mentioned results, PCA analysis showed that geographical location was the main factor shaping the composition of microbial communities. Although previous studies do show some evidence for biogeographic distribution patterns of microbial communities in the soils^76–78^, we are first to perform a medium-distance research on the biogeographic distribution of mangrove microbial communities on microbial communities in a type of a marine habitat, i.e. the mangrove ecosystem. The results of our study show that the mangrove microbial communities were distributed according to biogeographic pattern, following a distance decay relationship.

In this work, a relatively broader variation of microbial communities was detected in Bei and Qin samples. We speculated that, besides geographic location, environmental conditions and ecological disasters are other two important factors that shape the microbial communities in the mangrove ecosystems of Guangxi province. Our results are similar to several previous findings. Jiang *et al*. studied the diversity and composition of the bacterial communities in sediments collected from four locations in Hong Kong, and found that rhizosphere effect of mangrove plants was significant in shaping the bacterial communities in mangrove sediments^34^. Furthermore, Varon-Lopez *et al*. analyzed the abundance, composition and diversity of sulfur-oxidizing (SOB) and sulfate-reducing bacteria (SRB) in sediments from one oil contaminated, one urban-waste- and sludge-contaminated and one pristine mangrove habitat in Brazil, finding a significant difference in microbial SOB and SRB communities between mangrove sediments of contaminated and uncontaminated mangrove swamps^79^. Equally important to note, Colares and Melo assessed the structure of microbial communities in sediments in the root zone of the red mangrove (*Rhizophora mangle*) at three different sites, and found that spatial distribution of microbial communities within the red mangrove habitats is controlled primarily by the abiotic variables of each habitat^80^. Regarding the results of our research, we speculate that variations in environmental conditions at the study sites may be the main reason causing the variation in microbial communities in some of the samples. Our speculation is based on the fact that the study sites with bigger variations in microbial communities were located in vicinity of a petrochemical plant, a human residential area or an aquatic farm (Table 1). However, further efforts and comprehensive studies on both environmental factors and microorganisms of mangrove swamps are required to elucidate the mechanisms underlying spatial distribution of microbial communities in mangrove ecosystems.

One of the tasks in the protection of mangrove habitats is to monitor and evaluate the healthiness at the ecosystem level. This task, however, is difficult to satisfy because of the highly complexity of mangrove ecosystem, especially the microbial communities in mangrove sediments. Evaluation of the ecosystem healthiness at the ecosystem level is particularly necessary for mangrove habitats that suffered from anthropogenic influences. Presently, high-throughput sequencing based microbiomic analysis has become a powerful method for providing profound insights into the microbial community, its diversity and ecological functions of microbial microorganism in mangrove sediments^81^. Although, the sediment samples in the present work were only collected at six different mangrove habitats in the northern Beibu Gulf in China, our results have global significance for the research and management of mangroves. As described in Table 1, what is specific is that this region is in the early stages of industrial development, so we can see mangrove habitats with different ecosystem conditions suffering varying degrees of anthropogenic disturbances. Therefore, from the perspective of microbiome, our data is valuable for monitoring and evaluating how human activity impact mangrove habitats.
